# Opportunities to increase the attractiveness of the German Public Health Service as an employer: Laura Arnold

**DOI:** 10.1093/eurpub/ckac129.180

**Published:** 2022-10-25

**Authors:** L Arnold, F Hommes, L Kellermann, F Fischer, L Jung, A Mohsenpour, JM Stratil

**Affiliations:** 1 German Network of Young Professionals in Public Health, Berlin, Germany; 2 Applied Research and Transfer, Academy of Public Health Services, Duesseldorf, Germany

## Abstract

For decades, the public health service in Germany (PHS) experienced shortages of young professionals and challenges in recruiting qualified personnel. To sustainably counteract this challenge, it is necessary to understand the reasons of the perceived low attractiveness of PHS as a potential employer among students and young professionals. Two cross-sectional online surveys were conducted to assess the attitudes of medical, public health, and health science students towards the PHS as a potential employer. Wave 1, conducted from 2019-12 to 2020-04, focused on factors associated with high and with low interest in working for the PHS. Wave 2 was conducted from 2020-06 to 2020-09 to capture changes that may have resulted from the newfound attention of the PHS during the SARS-CoV-2 pandemic. Participants in both waves were asked about opportunities to increase the attractiveness of the PHS, which were analyzed using qualitative content analysis. In total 3040 students participated. Low interest in the PHS was associated with limited knowledge about public health, primary interest in clinical medicine, and a negative image of the public service. The qualitative analysis indicated as major obstacles: low visibility of and low awareness about the PHS, a perception of hierarchical and bureaucratic workplaces, and perceptions of repetitive occupations, among others. The participants suggested: improving awareness about the PHS in the population, including PHS in curriculum, and reducing entry barriers for non-medical students. The results of the largest survey of students on the attractiveness of the ÖGD in Germany provide valuable insights for ongoing reform processes. In addition to approaches to increase external visibility, existing processes and procedures within the ÖGD should be considered.

**Key messages:**

• To counteract the shortage of skilled workers, the PHS must become more attractive to young professionals. This requires both, greater external visibility and modernization of internal structures.

• Successful inclusion of the perspective of young professionals in the current modernization processes introduces opportunities to increase the attractiveness of the PHS in the long-term.

